# Rapid Detection of Extensively Drug-Resistant Tuberculosis in Clinical Samples Using a Novel Tabletop Platform: Protocol for a Prospective Clinical Study

**DOI:** 10.2196/26748

**Published:** 2021-07-14

**Authors:** Naomi Hillery, Marva Seifert, Donald G Catanzaro, Symone McKinnon, Rebecca E Colman, Peter G Chiles, Dumitru Chesov, Nelly Ciobanu, Christopher Hagan, Valeriu Crudu, Antonino Catanzaro, Timothy C Rodwell

**Affiliations:** 1 University of California, San Diego La Jolla, CA United States; 2 University of Arkansas Fayetteville, AR United States; 3 Nicolae Testemitanu State University of Medicine and Pharmacy Chisinau Republic of Moldova; 4 Division of Clinical Infectious Diseases Research Center Borstel Borstel Germany; 5 Institute of Phthisiopneumology “Chiril Draganiuc” Chisinau Republic of Moldova

**Keywords:** tuberculosis, drug-resistant, extensively drug-resistant, diagnostic, rapid treatment methods, protocol, drug susceptibility testing, prospective cohort study

## Abstract

**Background:**

The lack of accurate and efficient diagnostic devices for extensively drug-resistant tuberculosis (XDR-TB) makes it a severe threat to global public health. A prospective clinical study in an intended-use cohort was designed to evaluate the Akonni Biosystems XDR-TB TruArray and lateral flow cell (XDR-LFC) to address this gap in tuberculosis diagnostics.

**Objective:**

This paper presents the protocol for a study that aims to document the conceptualization and design of this evaluation method for early dissemination while data collection and analysis are ongoing.

**Methods:**

The clinical study was conducted in three phases. The first phase was to observe changes in bacterial load and culture positivity in patient sputa over time and better understand the diversity of prospective clinical samples. The second phase was to prospectively collect clinical samples for sensitivity and specificity testing of the Akonni Biosystems XDR-LFC device. Lastly, the third phase was to explore the anti-TB drug concentrations in serum throughout the drug-resistant tuberculosis treatment.

**Results:**

The methodology described includes the study design, laboratory sample handling, data collection, and the protection elements of human subjects of this clinical study to evaluate a potential new XDR-TB diagnostic device. A total of 664 participants were enrolled across the three phases. The implemented complex systems facilitated a thorough clinical data collection for an objective evaluation of the device. The study is closed to recruitment. The follow-up data collection and analysis are in progress.

**Conclusions:**

This paper outlined a prospective cohort study protocol to evaluate a rapid XDR-TB detection device, which may be informative for other researchers with similar goals.

**International Registered Report Identifier (IRRID):**

DERR1-10.2196/26748

## Introduction

### Background

Regional increases in the prevalence of drug-resistant tuberculosis (DR-TB) pose a significant threat to global tuberculosis (TB) control [1,2]. The World Health Organization (WHO) defined extensively drug-resistant tuberculosis (XDR-TB) as multidrug-resistant (MDR) or rifampicin-resistant (RIF-R) tuberculosis strains that are also resistant to fluoroquinolone (FQ) and either bedaquiline or linezolid (or both) [3]. XDR-TB has been associated with up to 80% mortality and is considered virtually untreatable in many parts of the world [1,4]. In 2018, the WHO estimated that there were approximately half a million new cases of DR-TB; however, only about 37% of those were detected and reported due to scarcity of rapid, efficient, and cost-effective solutions for detecting the resistance [1].

Early and rapid DR-TB detection and treatment with appropriate drugs are the essential effective control strategies to reduce DR-TB transmission and improve treatment outcomes. The 2020 WHO Global TB Report highlights that a pillar of the End TB Strategy and the United Nations (UN) Sustainable Development Goals (SDGs) requires intensified effort toward major technological developments by 2025, including rapid point-of-care tests for detecting drug resistance [5]. Current phenotypic drug susceptibility testing (pDST) can take up to 16 weeks to complete [6]. If not detected and treated rapidly, the continued XDR-TB transmission can cause massive disruptions to health care systems, economies, and lives on a local, national, and global scale. Unfortunately, despite the increasing global prevalence of XDR-TB, there are still no US Food and Drug Administration (FDA)–approved diagnostic platforms for the rapid diagnosis of XDR-TB, leaving individuals vulnerable to potential exposure.

There is a critical need for a rapid, highly sensitive, and specific tabletop platform to diagnose XDR-TB from patient samples directly. This clinical study was designed to combine the experience, resources, and existing diagnostic testing capabilities of the Global Consortium for Drug-resistant Tuberculosis Diagnostics (GCDD) (NIAID U01AI082229) with the technological innovation and industry knowledge of Akonni Biosystems to evaluate a rapid XDR-TB detection platform based on the detection of resistance-conferring mutations. During the initial design and development stage, the research team expanded and validated an existing prototype, the gel element microarray (GEM) platform (NIAID RC3 AI089106 and R43 EB011274), to detect clinically relevant single nucleotide polymorphisms that confer resistance in XDR-TB strains (NIAID R01AI111435). This paper describes the design and methods of a clinical study for evaluating the Akonni Biosystems XDR-TB TruArray and lateral flow cell (XDR-LFC) (Fredrick).

### Study Setting

The Republic of Moldova is a former Soviet republic located between Romania and Ukraine with a population of approximately 4 million people [7]. Its capital, Chisinau, is the most densely populated city, with about 640,000 residents [7]. The WHO ranks Moldova as one of the top 10 countries with the highest global multidrug-resistant tuberculosis (MDR-TB) burden [8]. The country reported approximately 24% new TB cases and 61% MDR-TB cases (previously treated with first-line drugs) in 2018 [8]. The Moldovan National TB Reference Laboratory (NRL) of the Phthisiopneumology Institute (PPI), Chisinau, has a staff of 25 and processes about 25,000 acid-fast bacilli (AFB) smears, 10,000 Xpert tests, 30,000 *Mycobacterium tuberculosis* (*Mtb*) cultures, and 10,000 culture-based pDST per year. Additionally, the NRL manages three regional TB laboratories and a sputum courier system that transports samples to and from these regional laboratories. The PPI was one of the three international laboratories that participated in the GCDD trial, and instituted a formal laboratory validation for relevant TB laboratory tests to ensure strict adherence to laboratory controls. Their previous research collaboration, along with high rates of DR-TB, made Moldova an ideal setting for the study in an intended-use population.

### Study Purpose and Aims

This study was designed primarily to determine the accuracy of the Akonni XDR-LFC device for detecting XDR-TB in an intended-use cohort. Participants were enrolled in three distinct but complementary phases at the Chisinau Municipal Hospital and regional TB treatment centers in the Republic of Moldova from 2014 to 2019, and were followed up for 2 years. Phase 1 involved exploring the changes observed in a TB bacterial load and culture positivity in patient sputa over time to understand the diversity of possible clinical samples, and validating the patient recruitment methods, laboratory processing procedures, and data collection instruments. In phase 2, we enrolled patients and collected prospective clinical samples from patients at risk for DR-TB to evaluate the sensitivity and specificity of the XDR-LFC instrument for detecting XDR-TB directly in clinical samples compared to reference DST. After the study initiation, an additional aim was included to explore the anti-TB drug concentrations in serum over the DR-TB treatment course and to provide preliminary data for future studies on the patient treatment response to the DR-TB treatment regimens.

## Methods

### Protocol Design

Phase 1 of the study was a prospective cohort study with serial sputum sampling. The sputum samples were collected from participants at the medical facilities daily on days 2-14, weekly on days 21, 28, and monthly on days 56, 84, 112, 140, and 168. At each sputum collection encounter, the study staff conducted a brief interview with the patient and a clinical assessment. Medical record data abstraction also occurred at these time points and an image of the directly observed therapy (DOT) record was collected.

Phase 2 was a prospective cohort study with a 24-month follow-up to assess a patient’s TB status and treatment outcomes. The sputum and blood samples were collected at the enrollment visit, along with the participant’s interview and medical record data abstraction. The patient records in the national TB registry will be reviewed at 24 months postenrollment to document the patient outcomes according to the WHO definitions (cured, completed, dead, failed, defaulted, and transferred) [8], and will be completed for all participants in 2021.

The eligible phase 2 participants were invited to enroll for the “phase 2+” arm of the study to evaluate the anti-TB drug concentrations in serum. Participants who consented to phase 2+ were followed up at day 7, 14, 28, 56, 84, 112, 140, and 168. At each time point, blood and sputum were collected, a brief patient interview and clinical assessment were conducted, and the medical record data, including an image of the DOT record, was abstracted and collected.

Previously published studies have utilized similar serial sample collection methods, and the outcome reviews aligned with the WHO treatment outcome guidelines [9,10]. The trial was not registered with ClinicalTrials.gov because this study was a prospective observational cohort study and did not meet the criteria for randomized controlled trials.

### Eligibility Criteria

Each phase had independent inclusion and exclusion criteria (Table 1). No phase included institutionalized or incarcerated subjects or pregnant women. Pregnant women were excluded because the medications required by the Moldovan National TB Program are not approved for use in pregnancy. Institutionalized and incarcerated subjects were excluded because the study was designed to evaluate this device in a general population and was not designed to assess the unique considerations of incarcerated or institutionalized populations. The study also required that the subjects produce at least 8.5 mL of sputum, a sample amount sufficient for all study procedures, at the enrollment.

**Table 1:** Inclusion and exclusion criteria by study phase.

Criteria	Phase 1	Phases 2 and 2+
Inclusion criteria	≥18 years of age RIF^a^ resistant by the GeneXpert *Mtb*^b^/RIF assay Enrolled within 1 week of RIF resistance determination by GeneXpert Not treated for TB^c^ for at least 4 weeks Intend to remain in Moldova for 24 months	≥5 years of age Suspected or confirmed clinically active TB disease (at least one of the following): AFB^d^ sputum smear positive within prior 7 days GeneXpert positive within prior 7 days Clinical suspicion of TB And suspected or confirmed DR-TB^e^ (defined as at least one of the following): Received >1 month of treatment for a prior TB episode Suspected of failing standard TB treatment Close contact with a known DR-TB case Diagnosed with RIF-R^f^ within the past 30 days Previously diagnosed with MDR-TB^g^ and suspected of failing a standard MDR-TB treatment regimen
Exclusion criteria	Pregnant women Institutionalized or incarcerated patients Patients unable to produce 8.5 mL of sputum for the study testing	Pregnant women Institutionalized or incarcerated patients Patients unable to produce 8.5 mL of sputum for the study testing Started treatment for the current TB episode more than 14 days prior to the enrollment date

^a^RIF: rifampin.

^b^*Mtb*: *Mycobacterium tuberculosis*.

^c^TB: tuberculosis.

^d^AFB: acid-fast bacilli.

^e^DR-TB: drug-resistant tuberculosis.

^f^RIF-R: rifampin resistant.

^g^MDR-TB: multidrug-resistant tuberculosis.

### Recruitment

Moldova’s national online TB registry, SIME-TB, was utilized to recruit potential participants for both primary phases of the study. The registry contains demographic data of all newly diagnosed TB cases. GeneXpert MTB/RIF devices were available at microscopy centers across Moldova, and the results were entered in SIME-TB within 1 to 4 days. All RIF-R patients were directed to go to one of the four regional TB clinics: 2 in Chisinau, 1 in Balti, and 1 in Vorniceni. The study physicians reviewed the SIME-TB and the patient intake logs for newly admitted patients to these TB clinics daily to identify patients suspected of suspected of TB or DR-TB. Once the potential participants were identified, the study staff approached the patients for screening, informed consent, and enrollment.

### Samples and Testing

As described in the Protocol Design section, the sputum and blood samples were collected at specified time points during each study phase. A minimum sputum volume was set for enrollment sample collection to ensure sufficient samples for all sputum tests. If a patient could not produce an 8.5 mL sputum sample initially, they were requested to try again in 2 hours following the first attempt; the samples were then pooled and measured again. Earlier experience with the GCDD trial showed that most patients could produce an 8.5 mL sputum sample required for the study. The follow-up sample collection did not have the minimum volume requirement.

The standardized procedures for processing the patient samples for testing are described below. Detailed reference figures were included in the study protocol to ensure a high degree of consistency. Solid culture using the Lowenstein–Jensen (LJ) medium and liquid culture using mycobacteria growth indicator tube 960 (MGIT 960) (Becton, Dickinson and Company) were performed using the validated protocols recommended by the WHO [11], consistent with the National TB Program (NTP) standards [11], and the manufacturer’s instructions [12]. Samples of *Mtb* DNA were extracted for shipment to the University of California, San Diego (UCSD), for next-generation sequencing (NGS).

### Phase 1

**Figure 1:**
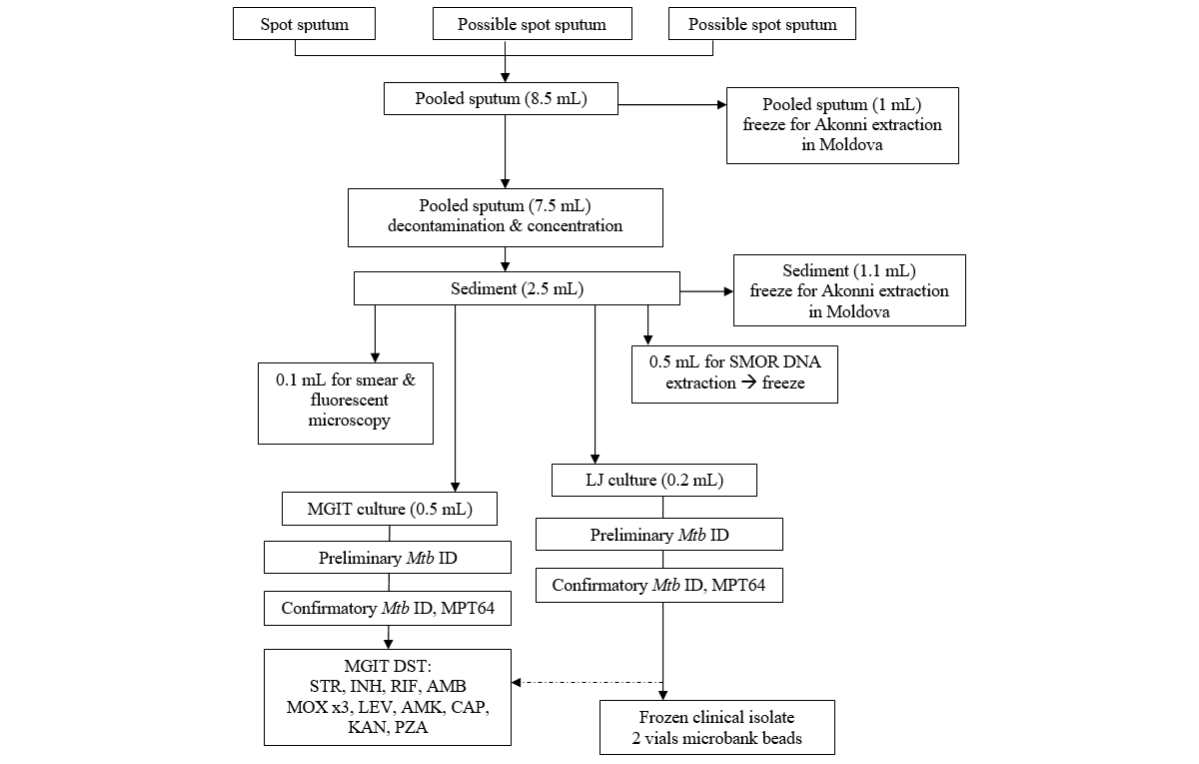
The phase 1 enrollment sample processing flowchart. AMK: amikacin; CAP: capreomycin; INH: isoniazid; KAN: kanamycin; LEV: levofloxacin; LJ: Lowenstein–Jensen; MGIT: mycobacteria growth indicator tube; MOX: moxifloxacin; *Mtb*: *Mycobacterium tuberculosis*; PZA: pyrazinamide; RIF: rifampin; SMOR: single molecule-overlapping read; STR: streptomycin.

Figure 1 shows the standardized flowchart of the day 1 (enrollment) sputum sample (≥8.5 mL) division and processing. The collected sputum sample was processed as follows:

- Raw sputum was processed to sediment (2.5 mL) a subsample of raw sputum
  - AFB smear was performed (0.1 mL)
  - A subsample of the sediment was frozen for later field testing by Akonni LFC (1.1 mL)
  - DNA was extracted from the sediment for shipment to the UCSD for NGS (0.5 mL)
- Solid LJ culture (0.2 mL; put on beads and frozen after growth)
- MGIT liquid culture (0.5 mL)
- MGIT DST reference testing was performed on confirmed *Mtb* positive specimens

Table 2 displays the prioritization of testing for sputum samples <8.5 mL at the follow-up. The follow-up sputum samples (maximum 20 per participant) were processed as follows:

- Raw sputum was processed to sediment
- AFB smear was performed
- A subsample of the sediment was frozen for later field testing by Akonni LFC
- Crude heat lysis extraction from the sediment for shipment to the UCSD for NGS
- Solid LJ culture (put on beads and frozen after growth)

**Figure 2:**
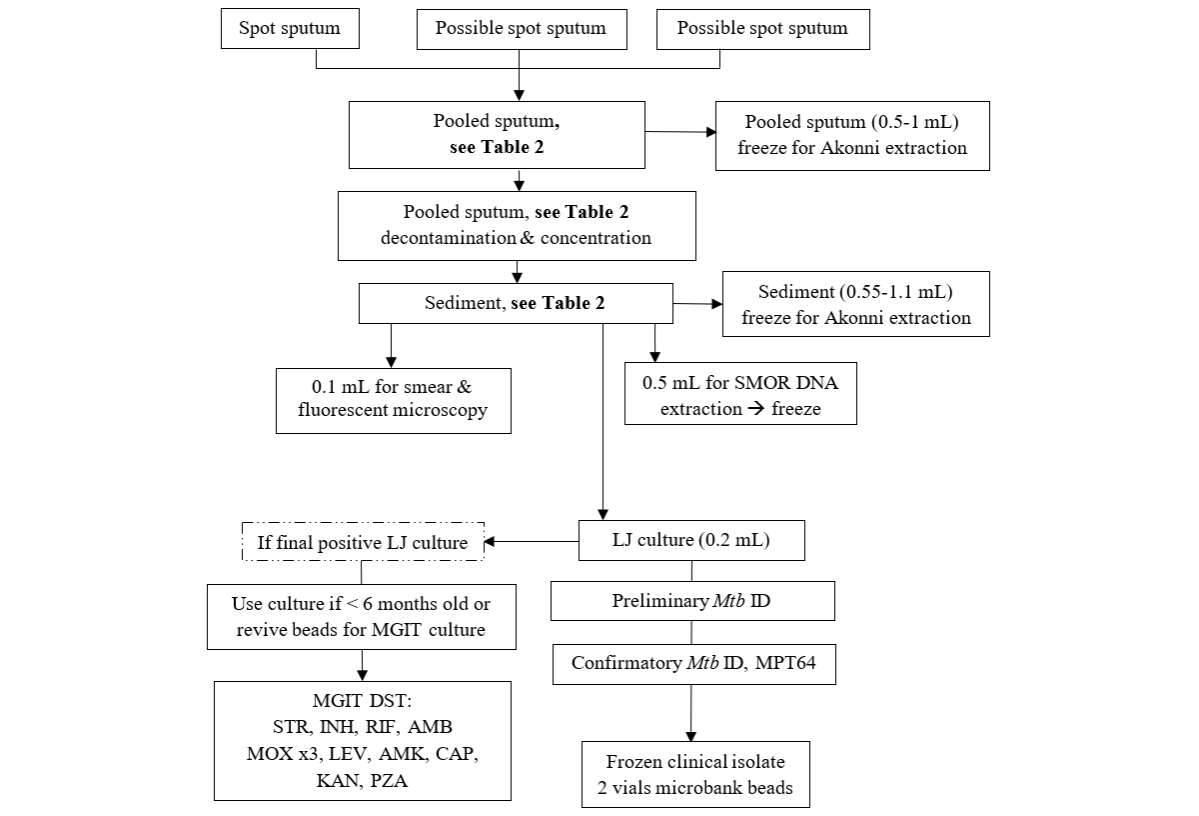
Phase 1 follow-up sputum sample processing schema. AMB: ethambutol; AMK: amikacin; CAP: capreomycin; INH: isoniazid; KAN: kanamycin; LEV: levofloxacin; LJ: Lowenstein–Jensen; MGIT: mycobacteria growth indicator tube; MOX: moxifloxacin; *Mtb*: *Mycobacterium tuberculosis*; PZA: pyrazinamide; RIF: rifampin; SMOR: single molecule-overlapping read; STR: streptomycin.

Figure 2 shows the standardized flowchart of the procedures used to ensure consistent sample processing. The first sputum and last culture positive sputum collected in phase 1 underwent DST using the MGIT 960 following the 2012 WHO recommendations for critical concentrations [11]: INH 0.1 μg/mL; RIF 1.0 μg/mL; ethambutol (AMB) 5.0 μg/mL; pyranzinamide (PZA) 100 μg/mL; KAN 2.5 μg/mL; AMK 1.0 μg/mL; CAP 2.5 μg/mL; levofloxacin (LEV) 1.5 μg/mL; and moxifloxacin (MOX) at 0.25 μg/mL, 0.5 μg/mL, and 2.0 μg/mL.

**Table 2:** Prioritization of testing for sputum samples <8.5 mL at the follow-up visits.

Pooled sputum	Sputum frozen	Sediment	Sediment frozen	AFB^a^ smear	SMOR^b^ DNA extract	LJ^c^ culture
>7.5 (mL)	1.0	2.5	1.1	0.1	0.5	0.2
6.5-7.4 (mL)	0.5^d^	2.0^d^	1.1	0.1	0.5	0.2
5-6.4 (mL)	0^e^	1.5^d^	0.55^d^	0.1	0.5	0.2
<4.9 (mL)	0^e^	1^d^	0^e^	0.1	0.5	0.2

^a^AFB: acid-fast bacilli.

^b^SMOR: single molecule-overlapping read.

^c^LJ: Lowenstein–Jensen.

^d^Reduced volume for the procedure.

^e^The procedure was skipped.

### Phase 2

The first 54 participant specimens underwent standard testing and had both raw sputum and sediment samples evaluated using the Akonni XDR-LFC device. A comparison of the LFC results from the raw sputum and the sediment guided the decision about which sample to use for LFC analysis for the remaining specimens. The raw sputum was used for the remainder of the samples and standard testing.

#### Phase 2 Standard Testing

**Figure 3:**
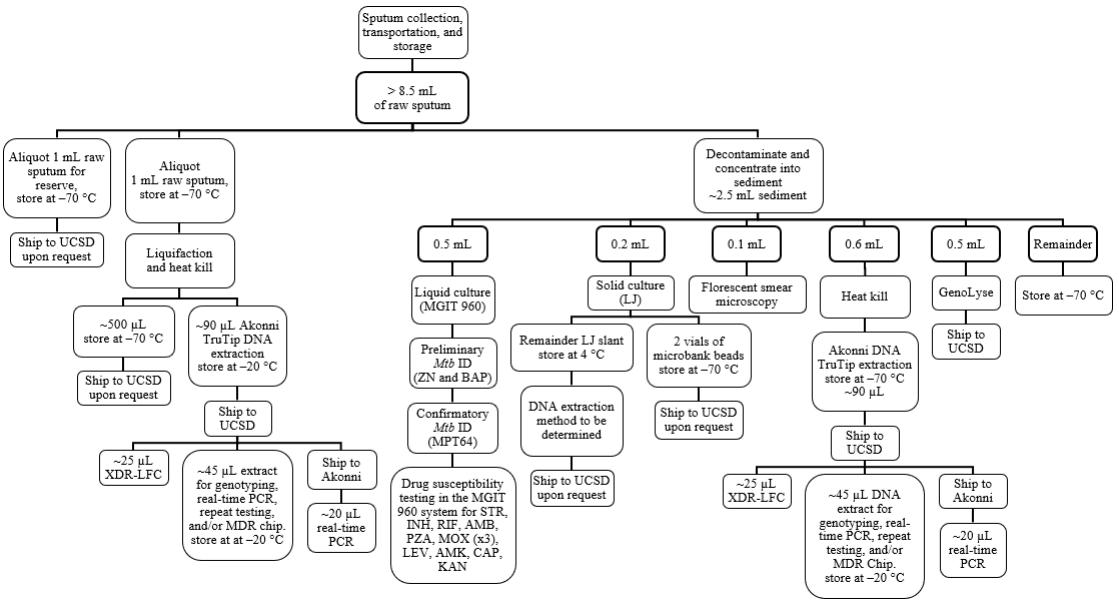
Phase 2 specimen flowchart for the first 54 participant samples. AMB: ethambutol; AMK: amikacin; CAP: capreomycin; INH: isoniazid; KAN: kanamycin; LEV: levofloxacin; LJ: Lowenstein–Jensen; MGIT: mycobacteria growth indicator tube; MOX: moxifloxacin; *Mtb*: *Mycobacterium tuberculosis*; PCR: polymerase chain reaction; PZA: pyrazinamide; RIF: rifampin; SMOR: single molecule-overlapping read; STR: streptomycin; UCSD: University of California, San Diego.

The day 1 (enrollment) sputum sample was divided as follows (Figure 3):

- For a subsample of raw sputum
  - Extracted DNA was frozen or run in real time with Akonni XDR-LFC (1 mL)
  - Raw sputum was kept in reserve for repeats or future testing (1 mL)
- Raw sputum was processed to sediment (~2.5 mL)
  AFB smear was performed
  Extracted DNA was frozen or run in real time with Akonni XDR-LFC
  Hain GenoLyse extraction DNA was batched and shipped to the UCSD for NGS
  Solid LJ culture (put on beads and frozen after growth)
  MGIT liquid culture
- *Mtb* confirmation testing was done
- MGIT DST (for INH, RIF, AMB, PZA, STR, KAN, AMK, CAP, LEV, and MOX at 3 concentrations) was performed on the confirmed *Mtb* positive specimens

The day 1 (enrollment) blood sample was collected in a 10-mL red-top tube, allowed to clot, centrifuged at 1300 g for 20 minutes to separate the serum, and frozen for storage at –70 °C.

#### XDR-LFC Device Procedure

The Akonni XDR-LFC device first extracts DNA from the heat-killed sputum using the Akonni TruTip workstation [13]. A laboratory technician transfers the extracted DNA to the XDR-LFC and places it in a polymerase chain reaction (PCR) thermocycler for DNA amplification and hybridization to specific molecular GEM probes printed on the XDR-LFC. After washing, the technician then places the XDR-LFC on the Akonni imaging device where the individual molecular GEM probes are illuminated. The illumination pattern characterizes the DNA signature of the sample (eg, wild type, single nucleotide polymorphisms, etc). The DNA extraction occurred at the PPI in Moldova on the TruTip workstation using extraction kits provided by the Akonni. A set of extracted DNA was run as a validation set on the XDR-LFC device in Moldova; however, all clinical samples used for analyses to date have been run at the UCSD.

### Phase 2+

**Figure 4:**
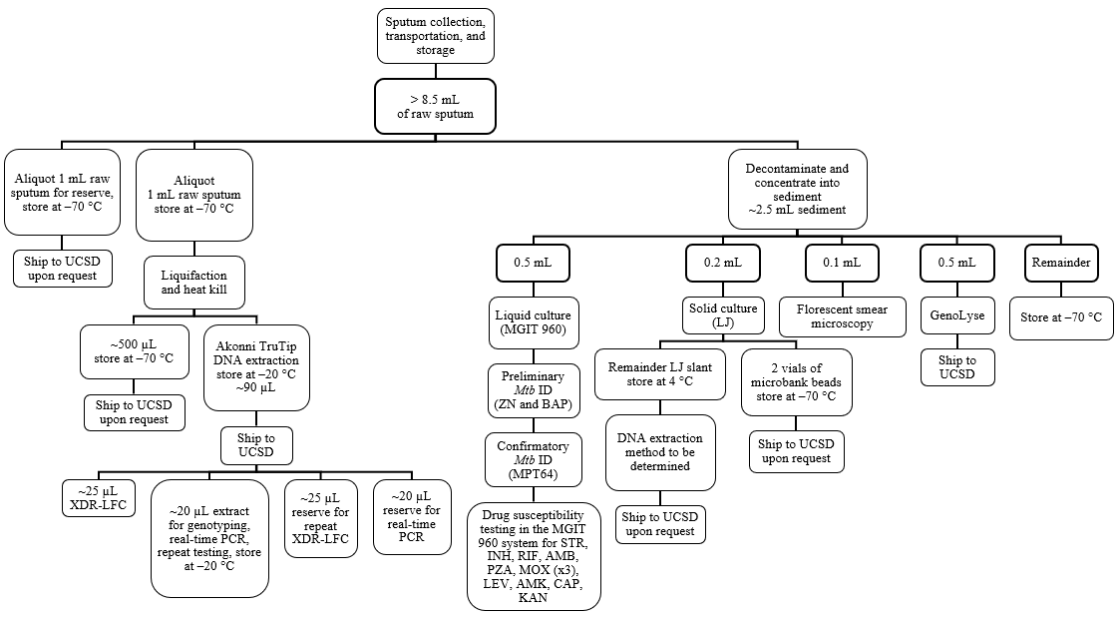
Phase 2 specimen flowchart for the remaining samples. AMB: ethambutol; AMK: amikacin; CAP: capreomycin; INH: isoniazid; KAN: kanamycin; LEV: levofloxacin; LJ: Lowenstein–Jensen; MGIT: mycobacteria growth indicator tube; MOX: moxifloxacin; *Mtb*: *Mycobacterium tuberculosis*; PCR: polymerase chain reaction; PZA: pyrazinamide; RIF: rifampin; SMOR: single molecule-overlapping read; STR: streptomycin; UCSD: University of California, San Diego.

The day 1 (enrollment) and the follow-up samples were processed as follows (Figure 4):

- Raw sputum was processed to sediment
- AFB smear was performed
- Solid LJ culture (put on beads and frozen at –70 °C after growth)
- The remainder of the sediment was frozen at –70 °C

#### Phase 2+ Blood Samples

There were 2 possible options for the blood draw. Option 1 was to draw blood at each of the 8 follow-up visits, and 2 samples were drawn from each patient at specific postdose time points. Option 2 included drawing blood at 2 follow-up visits, and 5 samples were drawn from each patient at specific postdose time points. A measured amount (10 mL) of blood (red-top tube) was collected from each participant, and the serum was separated, aliquoted, and frozen at –70 °C following standard blood collection and processing procedures.

### Patient Data Collection and Management

The patient data for phases 1 and 2 of this study were collected through face-to-face interviews, medical record reviews, and laboratory procedure documentation. The data were entered electronically using a data capture system built with the web-based software, QualtricsXM (Qualtrics). The data were entered into online forms with tablets provided to the staff in Moldova. Paper forms as backup were available in the event of any technical error. The Qualtrics surveys allowed access to questionnaires during unreliable internet as well—data could be entered and saved, and subsequently uploaded when the internet was available. The network of questionnaires built for this study employed an authenticator within Qualtrics; this tool confirmed that the ID a clinician intended to enter data for was still active and reduced the likelihood of applying the wrong data to a study ID number. The study ID assignment utilized a prefix of BP-A or BP2- to differentiate the participants enrolled in phase 1 and phase 2, respectively. This system benefits both data management and laboratory sample management aspects of the study. The samples generated from the study were accompanied by a 2-letter suffix code to indicate the sample type.

Electronic data capture was also utilized for front-end validation of data, ensuring that the data could only be entered in an expected range or format. Once the data were received by the data team, they were reviewed for any missing values and internally or externally invalid responses. The data team followed up these issues on an ongoing basis with the study staff in Moldova. Corrections to the data were documented systematically in a data cleaning log, and a syntax was used to merge data into a final and complete data set for analysis.

### Research Ethics

The study was approved by the UCSD Human Research Protections Program (Project #161864), and the Ethics Committee of the PPI “Chiril Draganiuc.” The consent document translation was completed by the study staff in Moldova fluent in Romanian. The participants were compensated with an equivalent of US$10 per patient per visit for time and travel; the compensation was customary and allowable by local norms, as well as the UCSD and Moldova IRB requirements. All participants were assigned a unique study identifier; no personally identifiable data were documented on the study questionnaires. A document linking the patient’s name to the study ID was stored securely in locked study files for follow-up purposes only. These records were destroyed upon completion of data collection. The questionnaires were stored in a separate secure location at the PPI and were archived for at least 5 years. No identifiable data were shared outside of the research team. The results from the XDR-LFC were used for research purposes only, and because they were not run in real time, it was not possible to use these experimental results for clinical decision-making. This protected the participants from any potential misuse of the experimental results.

## Results

### Participant Characteristics

The study enrolled 25, 639, and 40 participants in phases 1, 2, and 2+, respectively. The 40 participants in phase 2+ were part of the 639 participants enrolled in phase 2. In each phase of the protocol, the primary outcomes of interest were culture status, smear status, and phenotypic drug susceptibility for the 10 study drugs (Table 3).

Additional data collected from the participants for epidemiological analyses included age, gender, race, ethnicity, marital status, income, education, previous treatment for TB, comorbid conditions (including HIV), social risk factors (drug use, prior incarceration, group housing, cigarette use, alcohol use), and geographical location at key time points in the TB episode. The clinical variables documented were height, weight, TB drugs used in previous treatment episodes, previous DST results, and prior culture results. Self-reported comorbid conditions were compared with the medical records.

**Table 3:** The primary outcome variables.

Variable		Description or concentration
**Bacteriological confirmation**		
	Culture	Solid or liquid culture
	AFB^a^ smear	AFB smear microscopy with grade
**MGIT^b^ 960 DST^c^ results (µg/mL)**		
	Isoniazid (INH)	0.1
	Rifampin (RIF)	1.0
	Ethambutol (AMB)	5.0
	Pyrazinamide (PZA)	100
	Streptomycin (STR)	1.0
	Kanamycin (KAN)	2.5
	Amikacin (AMK)	1.0
	Capreomycin (CAP)	2.5
	Levofloxacin (LEV)	1.5
	Moxifloxacin at 0.25 (MOX0.25)	0.25
	Moxifloxacin at 0.5 (MOX0.5)	0.5
	Moxifloxacin at 2.0 (MOX2.0)	2.0

^a^AFB: acid-fast bacilli.

^b^MGIT: mycobacteria growth indicator tube.

^c^DST: drug susceptibility testing.

### Molecular Assay Findings

A comparison of the LFC to reference standard pDST was made by calculating the sensitivity and specificity of the LFC and comparing it with the reference standard pDST results for the drugs under study. Similarly, a comparison of the LFC to genotypic DST was made by calculating the positive and negative percent agreement of the LFC and comparing it with the sequencing results of the study. The standard percent agreement calculations for both phenotypic and genotypic comparisons were assessed by the ability to correctly classify MDR-TB and XDR-TB. For the follow-up analyses, the culture conversion at 6 months will be assessed by mutation, class of drug, and resistance profile. Logistic regression will be used to account for the treatment regimens and other risk factors typically associated with poor treatment outcomes. When 24-month follow-up data become available, the survival curves will be calculated for each mutation, class of drug, and resistance profile (susceptible, MDR, XDR, etc) based on the LFC results, considering the risk factors commonly associated with TB mortality. In addition, using logistic regression, the contributions of specific mutations to poor treatment outcomes will be assessed, taking into account the risk factors typically associated with poor treatment outcomes at 24 months. A comparison of multiple cultures, smear, and molecular diagnostic techniques will be performed using the serially collected phase 1 sputum samples. The anti-TB drug concentrations in the serum over the course of treatment and the associated patient response to the treatment collected during phase 2+ will be analyzed subject to future funding. As of April 2021, this study is closed to recruitment. The follow-up data collection and analysis are in progress.

## Discussion

This paper aimed to disseminate the protocol for a prospective cohort study to evaluate a device for detecting XDR-TB rapidly. Future publications from this study will address these findings. The absence of an FDA-approved rapid diagnostic for XDR-TB diagnosis in the United States and the lack of a near-patient tabletop integrated solution worldwide necessitates accessibility to a highly sensitive and specific molecular assay for rapid detection of XDR-TB globally. Such a device and assay would significantly improve the treatment timelines and allow for more successful patient outcomes. We continue to analyze the study samples gathered, as they are an invaluable resource to evaluate new diagnostic devices as they become available.

## Abbreviations

AFB: acid-fast bacilli
AMB: ethambutol
AMK: amikacin
CAP: capreomycin
DOT: directly observed therapy
DR-TB: drug-resistant tuberculosis
DST: drug susceptibility testing
FDA: US Food and Drug Administration
FQ: fluoroquinolone
GCDD: Global Consortium for Drug resistant Tuberculosis Diagnostics
GEM: gel element microarray
INH: isoniazid
KAN: kanamycin
LEV: levofloxacin
LFC: lateral flow cell
LJ: Lowenstein–Jensen
MDR-TB: multidrug-resistant tuberculosis
MGIT: mycobacteria growth indicator tube
MOX: moxifloxacin
*Mtb*: *Mycobacterium tuberculosis*
NGS: next-generation sequencing
NRL: National TB Reference Laboratory
NTP: National TB Program
pDST: phenotypic drug susceptibility testing
PCR: polymerase chain reaction
PPI: Phthisiopneumology Institute
PZA: pyranzinamide
RIF: rifampin
RIF-R: rifampicin-resistant
STR: streptomycin
TB: tuberculosis
UCSD: University of California, San Diego
WHO: World Health Organization
XDR-TB: extensively drug-resistant tuberculosis
